# Tubulin glycylation controls ciliary motility through modulation of outer-arm dyneins

**DOI:** 10.1091/mbc.E24-04-0154

**Published:** 2024-07-01

**Authors:** Tomohiro Kubo, Rinka Sasaki, Toshiyuki Oda

**Affiliations:** a Department of Anatomy and Structural Biology, Graduate School of Medicine, University of Yamanashi, 1110 Shimokato, Chuo, Yamanashi, 409-3898, Japan; University of Massachusetts Medical School

## Abstract

Tubulins undergo several kinds of posttranslational modifications (PTMs) including glutamylation and glycylation. The contribution of these PTMs to the motilities of cilia and flagella is still unclear. Here, we investigated the role of tubulin glycylation by examining a novel *Chlamydomonas* mutant lacking TTLL3, an enzyme responsible for initiating glycylation. Immunostaining of cells and flagella revealed that glycylation is only restricted to the axonemal tubulin composing the outer-doublet but not the central-pair microtubules. Furthermore, the flagellar localization of TTLL3 was found to be dependent on intraflagellar transport. The mutant, *ttll3(ex5)*, completely lacks glycylation and consequently exhibits slower swimming velocity compared with the wild-type strain. By combining the *ttll3(ex5)* mutation with multiple axonemal dynein-deficient mutants, we found that the lack of glycylation does not affect the motility of the outer-arm dynein lacking mutations. Sliding disintegration assay using isolated axonemes revealed that the lack of glycylation decreases microtubule sliding velocity in the normal axoneme but not in the axoneme lacking the outerarm dyneins. Based on our recent study that glycylation occurs exclusively on β-tubulin in *Chlamydomonas*, these findings suggest that tubulin glycylation controls flagellar motility through modulating outer-arm dyneins, presumably by neutralizing the negative charges of glutamate residues at the C-terminus region of β-tubulin.

## Abstract (executive summary)

Tubulins undergo various posttranslational modifications (PTMs), including glycylation, but the role of this PTM in ciliary and flagellar motility remains unclear.

In this study, the authors generated a novel *Chlamydomonas* mutant lacking the monoglycylase TTLL3 to investigate the significance of tubulin glycylation in ciliary function. Their findings reveal that glycylated tubulin regulates ciliary motility by modulating outer arm dyneins.

These results highlight the substantial impact of glycylation on ciliary motility, potentially by neutralizing the negative charges on β-tubulin. This insight paves the way for further exploration of cellular motility mechanisms and their implications in diseases associated with ciliary dysfunction.

## INTRODUCTION

Alpha- and β-tubulin undergo several posttranslational modifications (PTMs) including acetylation, tyrosination, detyrosination, glutamylation, and glycylation (Westermann and Weber, 2003; Janke and Bulinski, 2011). These PTMs increase the functional diversity of microtubule-based cell organelles such as eukaryotic cilium (or flagellum), a hair-like appendage on cell surfaces. Because α- and β-tubulin constitute the major components of the ciliary axoneme, cilia provide a remarkable model for studying tubulin PTMs.

Glutamylation and glycylation take place at γ-carboxyl group of glutamate residues located near the tubulin C-termini (Eddé *et al.*, 1990; Mary *et al.*, 1996). They are catalyzed by enzymes belonging to the tubulin tyrosine ligase-like (TTLL) protein family (Janke *et al.*, 2005; Wloga *et al.*, 2009). In mammals, 13 types of TTLL proteins exist, with TTLL 1, 4, 5, 6, 7, 9, and 11 responsible for glutamylation (Dijk *et al.*, 2007; Konno *et al.*, 2016), and TTLL 3, 8, and 10 responsible for glycylation (Ikegami and Setou, 2009; Rogowski *et al.*, 2009; Wloga *et al.*, 2009). These modifications are likely to be reversible, as deglutamylases performing the removal of glutamylation have been identified as members of the cytosolic carboxypeptidase (CCP; Rogowski *et al.*, 2010) and tubulin metallocarboxypeptidase (TMCP; Nicot *et al.*, 2023). TTLLs, deglutamylases, and unidentified deglycylases should exhibit distinct specificities for both substrate and reaction activity, most likely resulting in the production of variable lengths of side chains for either polyglutamylation or polyglycylation.

The importance of tubulin polyglutamylation for ciliary function has been explored using several organisms. We and others have demonstrated that tubulin glutamylation affects ciliary motility (Ikegami *et al.*, 2010; Kubo *et al.*, 2010; Suryavanshi *et al.*, 2010). Through an investigation of *Chlamydomonas* mutant *tpg1* harboring a mutation in the gene encoding TTLL9, we have established that the negative charges of tubulin polyglutamylation modulates the activity of an axonemal structure called the nexin-dynein regulatory complex (N-DRC) (Kubo *et al.*, 2012; Alford *et al.*, 2016; Kubo and Oda, 2017). This concept gains further support from a recent structural study indicating that polyglutamylated tubulin is specifically localized to the protofilament 9 of the outer-doublet micro­tubules in both *Chlamydomonas* and mouse (Viar *et al.*, 2023). This location should enable a direct interaction between polyglutamylated tubulin and the N-DRC.

Tubulin glycylation for ciliary function has also been extensively studied. A study on *Tetrahymena* mutants possessing a mutation in the gene encoding TTLL3 revealed that tubulin glycylation plays a crucial role in regulating ciliary assembly (Wloga *et al.*, 2009). Similarly, glycylation was shown to be essential for the stability and maintenance of ependymal cilia (Bosch Grau *et al.*, 2013), as well as the control of primary cilia length (Gadadhar *et al.*, 2017). More recently, glycylation was found to be important for flagellar motility, as demonstrated in sperm analysis of *TTLL3^–/–^ TTLL8^–/–^* double-knockout mouse (Gadadhar *et al.*, 2021) and *Chlamydomonas* mutant lacking TTLL3 (Viar *et al.*, 2023). These studies suggest that glycylated tubulin significantly influences the function of the axonemal dyneins. However, the biochemical properties of TTLL3 and which dynein’s function is mostly affected by glycylation are still obscure. In this study, we generated *Chlamydomonas* strains lacking TTLL3 by a CRISPR/Cas9 mediated gene editing. By investigating several dynein-lacking mutants combined with the *ttll3* mutation, we found that the motilities of mutants lacking outer-dynein arm are not affected by tubulin glycylation. This result suggests that tubulin glycylation affects the function of outer-arm dynein. Our study thus shed light on a novel aspect of tubulin glycylation in cilia and flagella.

## RESULTS

### A novel *Chlamydomonas* mutant harboring an insertion in the TTLL3 encoding gene

**FIGURE 1:**
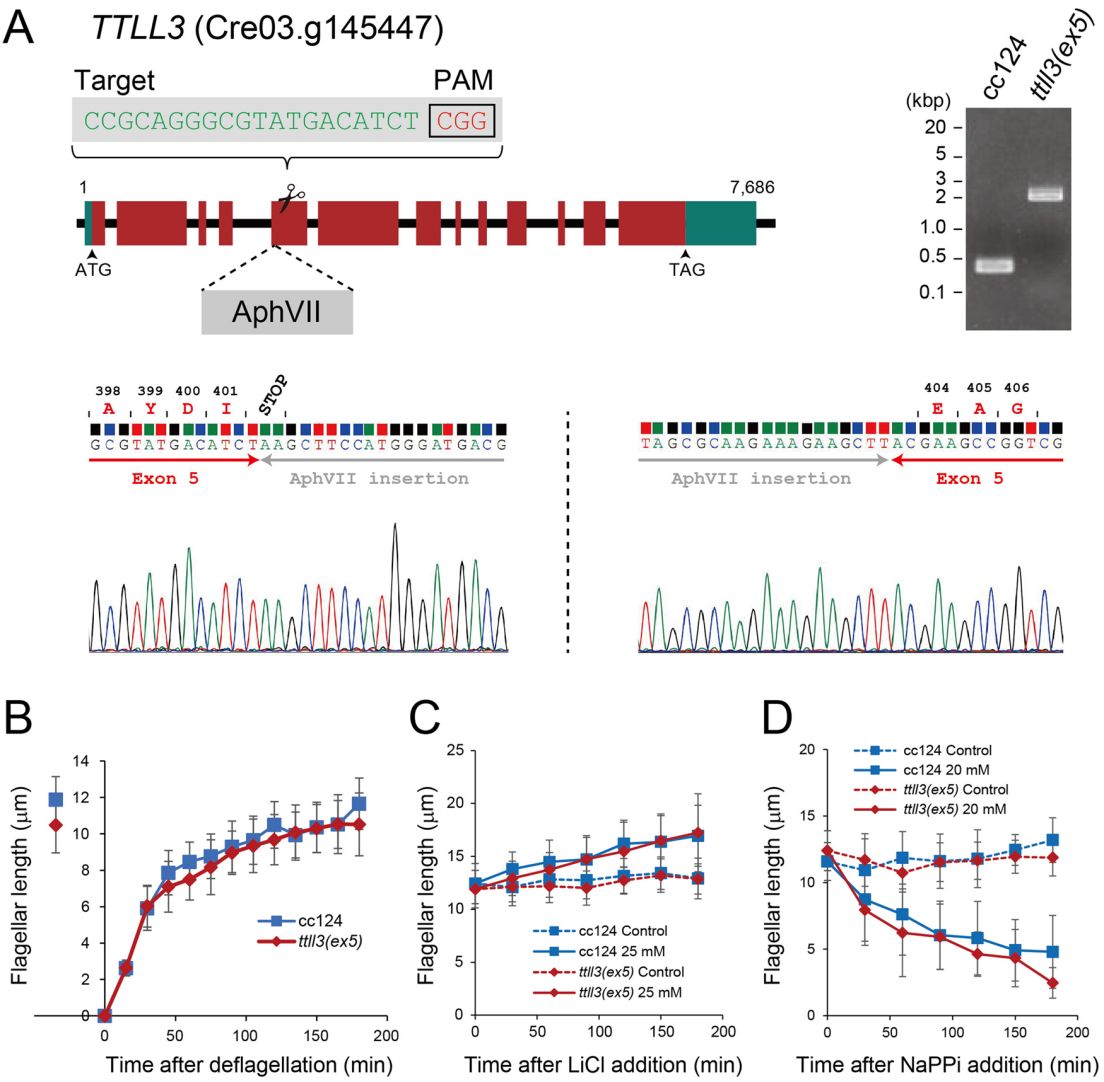
Generation of the *ttll3(ex5)* mutant (A) The structure of the *TTLL3* (Cre03.g145447) gene. Exons are indicated in dark red, and untranslated regions are shown in greenish blue. As confirmed by gene sequencing and PCR using a specific primer set, a hygromycin-resistant gene cassette (dark gray) was integrated into exon 5 of the *TTLL3* gene. (B) Flagellar regeneration kinetics of wild type (cc124) and *ttll3(ex5)*. Cells were deflagellated by pH shock, and flagellar lengths were measured every 15 min for up to 180 min. (C) Flagellar elongation of wild type (cc124) and *ttll3(ex5)* induced by 25 mM LiCl. (D) NaPPi-induced flagellar shortening of wild type (cc124) and *ttll3(ex5)*. In (B–D), at least 50 flagella were measured for each time points. The average values and standard deviations are shown.

In the *Chlamydomonas* genomic database, we found a gene encoding potential homologue of mouse TTLL3, a monoglycylase. Interestingly, among the glycylation enzymes TTLL3, TTLL8, and TTLL10 reported in other organisms, only TTLL3 is present in *Chlamydomonas*. The absence of TTLL10 responsible for elongation of polyglycylated chains likely explains the lack of polyglycylation in *Chlamydomonas* (Kubo *et al.*, 2023). To explore the role of TTLL3 in *Chlamydomonas*, we transformed the wild-type strain (cc124) by integrating a hygromycin-resistant cassette into the *TTLL3* gene (Cre03.g145447) using a CRISPR/Cas9-mediated gene editing (Figure 1A). Genotyping with a specific primer set identified several transformants with a hygromycin-resistant cassette inserted into exon 5 of *TTLL3* (Figure 1A). Gene sequencing revealed that several transformants have a premature stop codon in the exon 5, indicating that the transcription of full-length TTLL3 is hindered (Figure 1A). The TTLL3 protein and thereby tubulin glycylation were found to be missing in the flagella of this mutant as we will describe later. We, therefore, refer to this strain as *ttll3(ex5)*. Daily measurements of cell concentration showed that *ttll3(ex5)* has normal cell growth (Supplemental Figure 1), suggesting that *TTLL3* is not involved in the cell proliferation and survival.

Because tubulin polyglycylation was reported to affect ciliary assembly (Wloga *et al.*, 2009; Bosch Grau *et al.*, 2013; Gadadhar *et al.*, 2017), we were interested in the characteristics of flagellar assembly and disassembly in *ttll3(ex5)*. Initially, we found that the kinetics of flagellar regeneration is normal in this mutant (Figure 1B). Subsequently, we determined that flagellar elongation triggered by LiCl treatment is also unaffected (Figure 1C). Furthermore, we found that rate of flagellar shortening induced by NaPPi treatment remained normal (Figure 1D). Therefore, these results suggest that tubulin glycylation does not critically affect polymerization nor depolymerization of axonemal microtubules in *Chlamydomonas*.

The lack of the *TTLL3* gene may cause a defect in the intraflagellar transport (IFT), even though the flagellar regeneration kinetics is normal in *ttll3(ex5;* Figure 1C). To investigate the function of IFT in *ttll3(ex5)*, we examined the amounts of IFT-particle proteins in isolated *ttll3(ex5)* flagella. Our findings indicate that the flagellar levels of IFT172, IFT139, IFT81, and IFT57 are normal in the *ttll3(ex5)* mutant (Supplemental Figure 2A). Immunostaining of cells further showed that the localization of these IFT-particle proteins remains unchanged in *ttll3(ex5)* (Supplemental Figure 2B). These results confirm that the mutation in the *TTLL3* gene does not impact the function of IFT.

### The mutant *ttll3(ex5)* completely lacks axonemal tubulin glycylation

**FIGURE 2:**
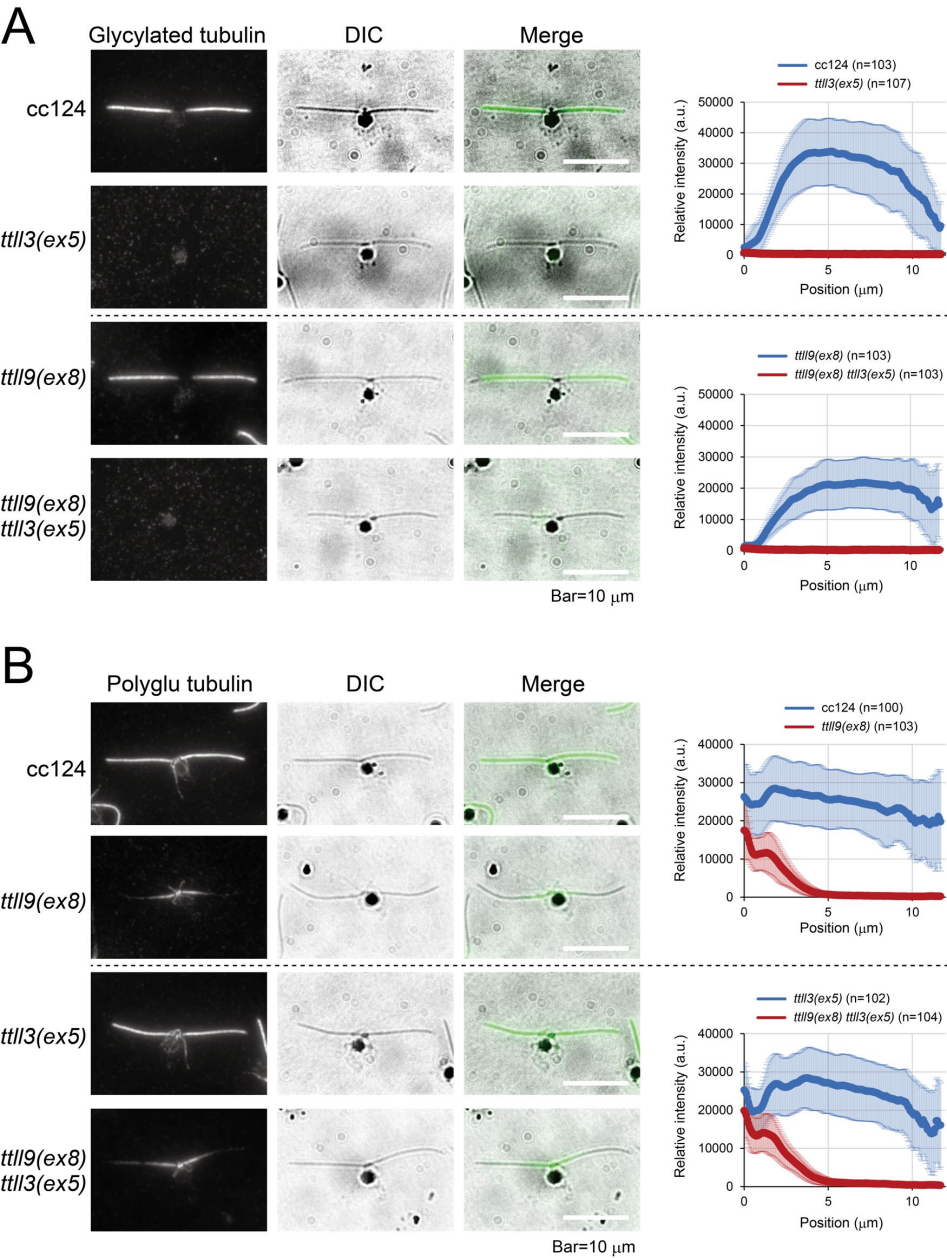
The *ttll3(ex5)* axoneme completely lacks tubulin glycylation Immunostaining of NFAp of wild type (cc124), *ttll3(ex5)*, *ttll9(ex8),* and *ttll3(ex5) ttll9(ex8)* using (A) antiglycylated tubulin (Gly-pep1) and (B) antipolyglutamylated tubulin (polyE#2) antibodies. One cilium each on at least 100 cells was investigated and the average signal intensities with standard deviations were obtained.

To investigate the level of glycylation in the cellular and axonemal microtubules, we performed indirect fluorescence microscopy of the nuclear-flagellar apparatuses (NFAps) using gly-pep1 antibody recognizing mono or bi-glycylated tubulin (Gadadhar *et al.*, 2017). In the wild-type axonemes, the gly-pep1 signal sharply increases towards the midsection, then progressively decreases towards the distal tips (Figure 2A). Interestingly, the staining was absent in the microtubules of the cytoplasm and the basal body, indicating that tubulin glycylation is only restricted to the axoneme. This differs from some ciliates such as *Paramecium* (Bré *et al.*, 1998) and *Tetrahymena* (Xia *et al.*, 2000) that have glycylated tubulin in the cytoplasmic microtubules. In contrast to the wild-type axoneme, however, the *ttll3(ex5)* axoneme exhibited a complete absence of the gly-pep1 staining (Figure 2A). This confirms that TTLL3 is the only monoglycylase expressed in *Chlamydomonas* (Viar *et al.*, 2023), and its defect causes complete loss of tubulin glycylation.

**FIGURE 3:**
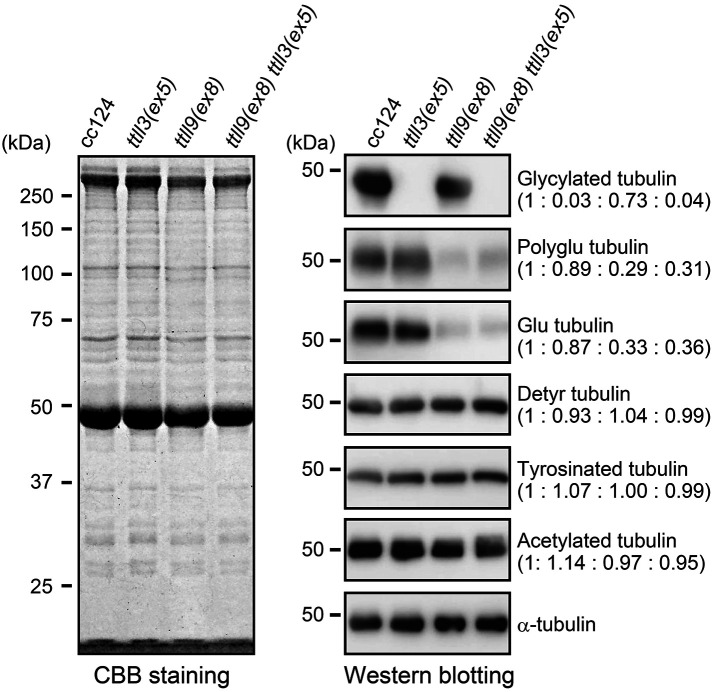
Amounts of modified tubulins in the *ttll3(ex5)* axoneme CBB-stained gel and Western blotting of cc124, *ttll3(ex5)*, *ttll9(ex8)*, and *ttll9(ex8) ttll3(ex5)* axonemes. The quantification of the band intensities, normalized to those of α-tubulin, is shown in brackets and is based on at least five experiments.

The levels of tubulin glycylation and polyglutamylation were believed to have an inverse correlation, as evidenced by studies showing that mutants lacking glycylation sites has increased polyglutamylation, and vice versa (Redeker *et al.*, 2005; Kubo *et al.*, 2023). To investigate whether a similar interplay exists in the *Chlamydomonas* TTLL mutants, we generated *ttll9(ex8)* strain deficient in the gene encoding TTLL9 glutamylase. Because we verified that the axonemes of *ttll9(ex8)* display greatly reduced polyglutamylation (Figures 2B and 3), this strain most likely is a null allele of *TPG1* locus (Kubo *et al.*, 2010). The decreased level of polyglutamylation, and consequently the reduced swimming velocity (Figure 7B), is identical to that observed in *tpg1*, a strain previously isolated (Kubo *et al.*, 2010). Surprisingly, we did not observe any increase in glycylation in the axonemes lacking glutamylation, nor did we detect any increase in glutamylation in the axonemes lacking glycylation (Figure 2, A and B). Instead, the novel *ttll9(ex8)* mutant showed slightly reduced level of glycylation in the axoneme (Figures 2, A and 3), and the *ttll3(ex5)* displayed slightly decreased or normal level of polyglutamylation in the axoneme (Figures 2B and 3). It appears that, at least in *Chlamydomonas*, the inverse correlation of these two modifications only takes place in strains that possess point mutations at the sites of modification on the C-terminus region of tubulin.

These results of the immunostaining were confirmed by Western blotting of isolated axonemes using several antibodies targeting modified tubulin (Figure 3). Our findings reveal a nearly complete absence of the gly-pep1 signal in the *ttll3(ex5)* axonemes. Additionally, we observed a reduction of polyglutamylation in the *ttll9(ex8)* axonemes. However, other PTMs of tubulin, such as acetylation, tyrosination, and detyrosination, remained almost unchanged in both the *ttll3(ex5)* and *ttll9(ex8)* axonemes. These results indicate that in *Chlamydomonas*, the loss of TTLL3 leads to a total loss of tubulin glycylation, while having a minimal effect on other tubulin modifications.

**FIGURE 4:**
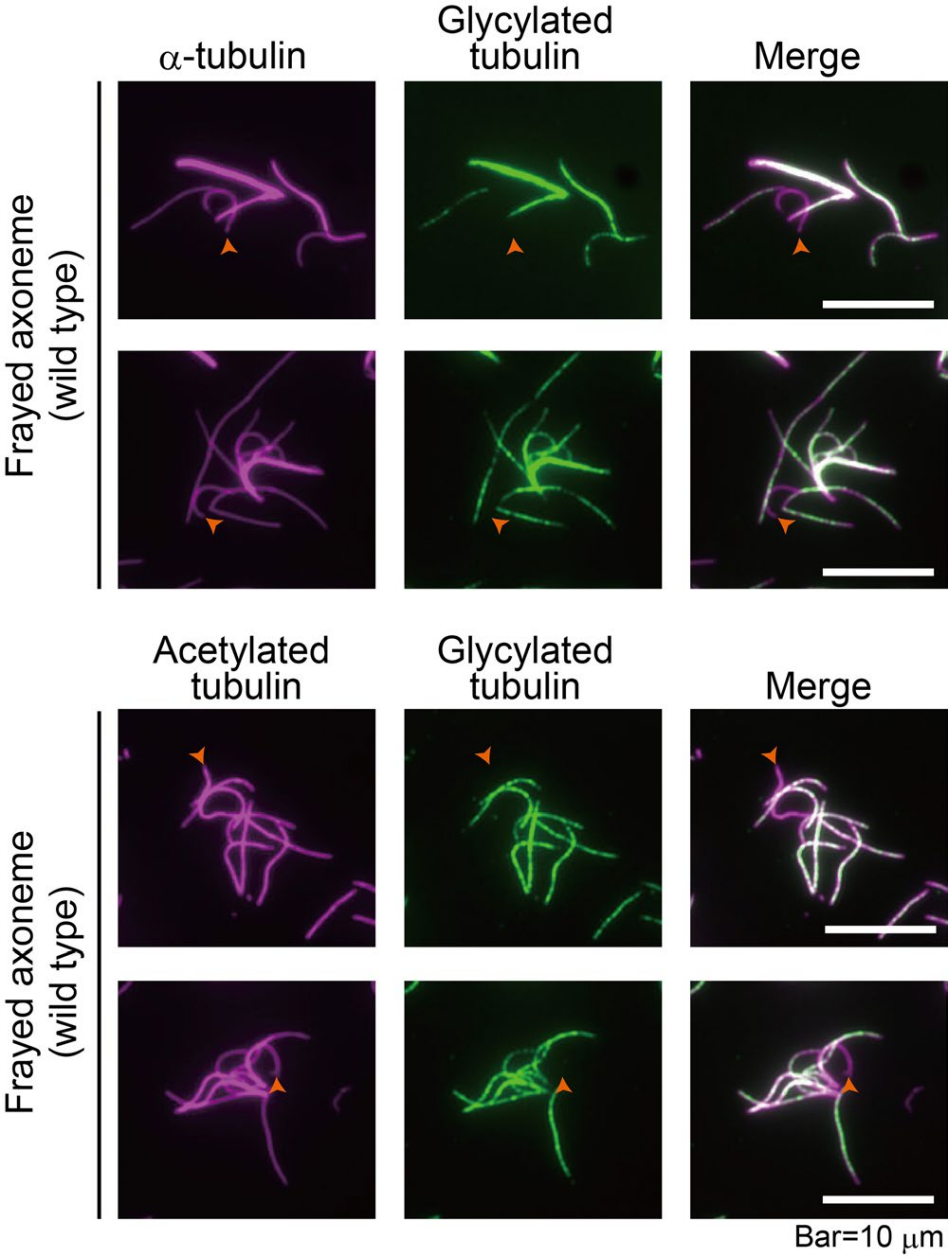
Glycylated tubulin is restricted to the outer-doublet microtubules Frayed axonemes were double stained with antibodies targeting the indicated tubulins. The positions of the central-pair microtubules were marked with orange arrowheads.

We then investigated whether tubulin glycylation is confined to specific axonemal microtubules. To accomplish this, we conducted double staining on frayed axonemes isolated from the wild-type strain using two sets of antibodies: one set with antibodies against α-tubulin and glycylated tubulin, and another set with antibodies against acetylated-α-tubulin and glycylated tubulin. We found that microtubule bundles of the outer doublets are stained by the gly-pep1 antibody. However, the central-pair microtubules, characterized by their strongly curved shape in contrast to the less curved outer doublets (Kamiya, 1982), are not stained by the gly-pep1 antibody (Figure 4). These observations contradict the findings of Orbach and Howard (2019), who stated that all axonemal micro­tubules including the central-pair microtubules are glycylated.

### Acquisition of tubulin glycylation on stable axonemal microtubules

**FIGURE 5:**
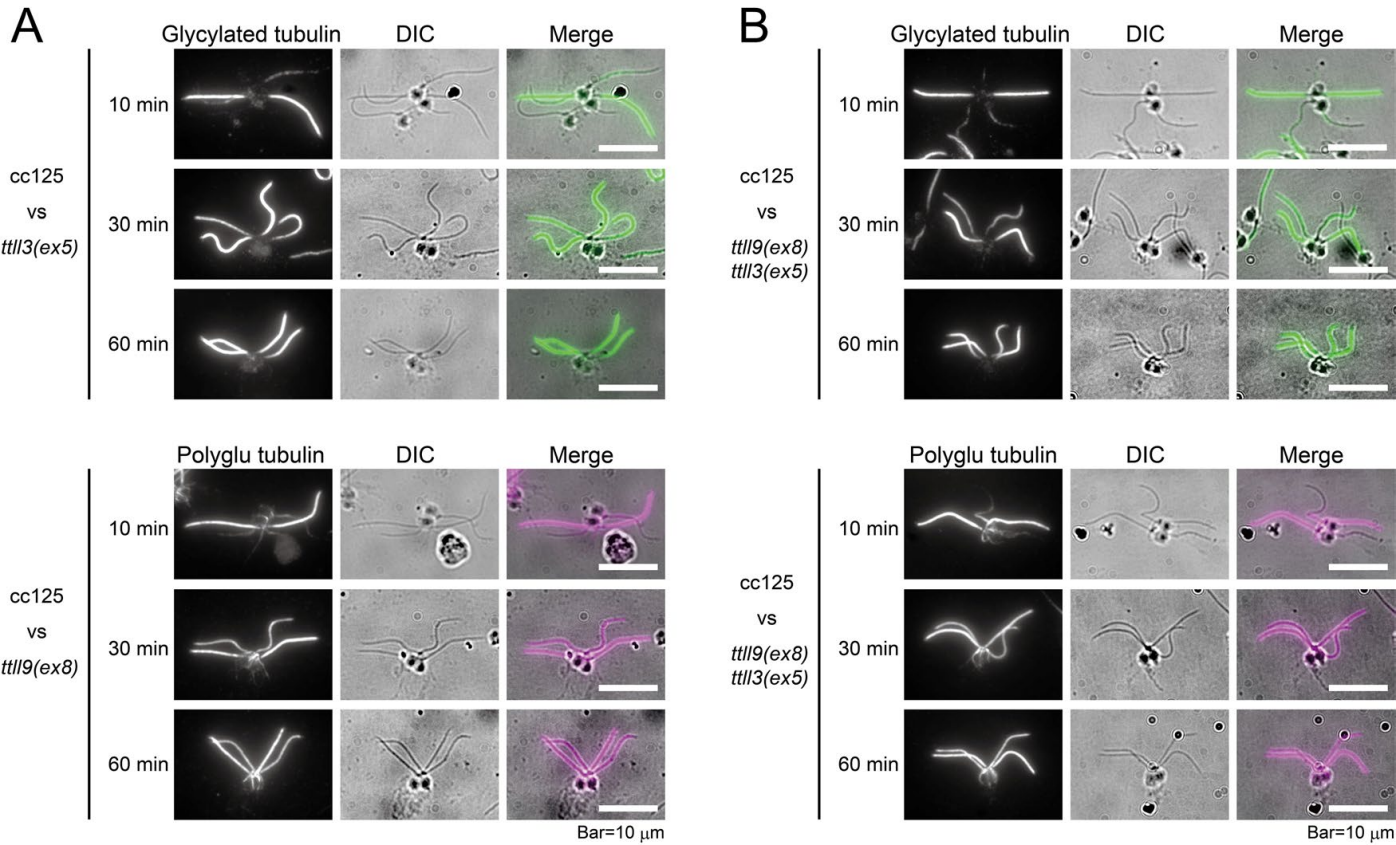
Recovery of tubulin glycylation on the axonemal microtubules (A) Immunostaining of the NFAp in dikaryons formed between wild type (cc125) and *ttll3(ex5)*, using the Gly-pep1 antibody (upper panel), or between cc125 and *ttll9(ex8)*, using polyE#2 antibody (lower panel). (B) Immunostaining of NFAp in dikaryons formed between wild type (cc125) and *ttll9(ex8) ttll3(ex5)* using the Gly-pep1 antibody (upper panel) or polyE#2 (lower panel)*.* Dikaryons were isolated at 10, 30, and 60 min after mixing the opposite mating types.

**FIGURE 6:**
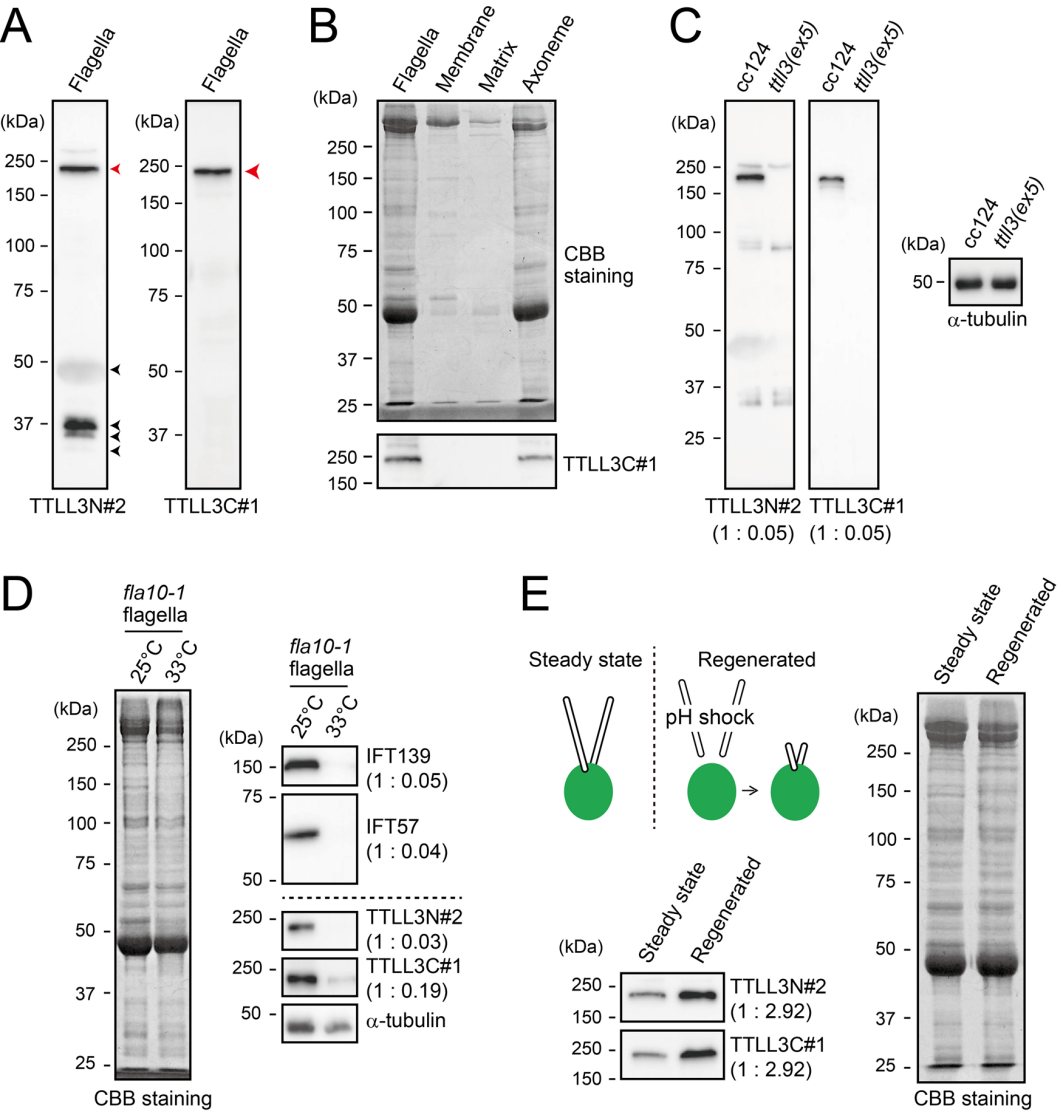
Flagellar localization of TTLL3 depends on the IFT (A) Characterization of peptide antibodies against the N-terminus and C-terminus of TTLL3. Flagellar proteins were subjected to Western blot analysis using anti-TTLL3N#2 (left panel) and TTLL3C#1 (right panel) antibodies. Red arrowheads indicate the specific bands for TTLL3, while black arrowheads indicate non-specific bands. (B) CBB staining (upper panel) and Western blot analysis (lower panel) of flagellar fractions. Most of the TTLL3 proteins is present in the axonemal fraction. (C) Western blot analysis of axonemes from wild-type (cc124) and *ttll3(ex5)* strains using anti-TTLL3N#2 and TTLL3C#1 antibodies. The quantification of the band intensities, normalized to those of α-tubulin, is shown in the brackets. (D) CBB-stained gel and Western blot analysis of flagella isolated from *fla10-1*, a temperature-sensitive mutant affecting IFT-dependent flagellar assembly, incubated with or without exposure to 33°C. The quantification of the band intensities, normalized to those of α-tubulin, is shown in the brackets. (E) Western blot analysis (left panel) and CBB staining (right panel) were performed on flagella from both steady-state and regenerated flagella. The quantification of the band intensities, normalized to those of tubulin intensities, is shown in the brackets.

The gametes of opposite mating types undergo cell fusion immediately after being mixed together, resulting a singular cell possessing four flagella and two nuclei. This specific cell type is called a “dikaryon” as described by Starling and Randall (1971). To investigate the process of glycylation on the stable axonemal microtubules, we undertook the mating of the wild-type (cc125) and *ttll3(ex5)* gametes to generate dikaryon cells. We assessed the dikaryons at time points 10, 30, and 60 min after the gamete mixing. Our observation revealed that the flagella of *ttll3(ex5)* underwent gradual acquisition of glycylation along the entire length within 30 min of cell fusion and nearly complete recovery within 60 min (Figure 5A). This pattern resembles the acquisition of tubulin polyglutamylation observed in the dikaryon between cc125 and *ttll9(ex8;* Figure 6A) as we previously reported (Kubo *et al.*, 2014).

We than produced dikaryons between cc125 and the double mutant *ttll9(ex8) ttll3(ex5)* to investigate whether the acquisitions of glycylation and glutamylation occur simultaneously. Immunostaining of dikaryons using gly-pep1 and polyE#2 antibodies revealed that the recovery of both glycylation and glutamylation occur gradually within 30 min (Figure 5B) with no noticeable delay compared with the dikaryons produced between cc125 and either *ttll3(ex5)* or *ttll9(ex8)*. These results indicate that both glycylation and glutamylation are a dynamic process that occurs on stable microtubules, and that both modifications do not interfere with each other.

### Axonemal localization of TTLL3 depends on the IFT

To study biochemical properties of TTLL3 in cells and flagella, we generated peptide antibodies targeting two distinct regions of TTLL3. Two antibodies against the N-terminal region (amino acids 4-24) were produced, referred to as anti-TTLL3N#1 and #2, respectively. Similarly, two antibodies against the C-terminal region (amino acids 1301–1319) were produced, referred to as anti-TTLL3C#1 and #2, respectively. When these antibodies were used for Western blot analysis of wild-type cytoplasm, no specific bands for TTLL3 were observed. Instead, several non-specific bands were detected (Supplemental Figure 3A). However, in Western blot of flagellar proteins, specific bands were detected in the range between 150 and 250 kDa with every antibody used (Figure 6A; Supplemental Figure 3B). Although the predicted molecular weight of TTLL3 is 146 kDa, the bands in the 150∼250 kDa range are likely to be signals of TTLL3. This is based on two key observations: (i) the specific bands at the same height are detected using two sets of antibodies targeting different amino-acid sequences, and (ii) the specific bands are not detected in the *ttll3(ex5)* axoneme as described below. The detection of specific bands exclusively in the flagella and not in the cytoplasm suggests that TTLL3 is predominantly enriched in the flagella. This concept is further supported by the observation that glycylation is confined to the axonemal microtubules but not to the cytoplasmic microtubules (Figure 2A). Despite the high specificity of these antibodies, however, the localization of TTLL3 proved challenging with both conventional immunofluorescence microscopy and confocal microscopy presumably due to its low expression level (unpublished data).

Western blotting of the flagellar fraction revealed that the majority of TTLL3 is localized to the axonemal fraction (Figure 6B). This property is identical to that of TTLL9, a tubulin polyglutamylase (Kubo *et al.*, 2010). Similar to TTLL9, TTLL3 might associate relatively strongly with the axonemal microtubules. Also, TTLL3 was found to be completely absent in the *ttll3(ex5)* axoneme (Figure 6C). This suggests that the mutant flagella do not contain truncated version of the TTLL3 protein, although a possibility remains that a shorter form is expressed in the cytoplasm of *ttll3(ex5)*.

To investigate whether the flagellar localization of TTLL3 depends on IFT, we studied the flagella of *fla10-1*, a temperature-sensitive mutant that affects IFT-driven flagellar assembly due to a point mutation in the kinesin-homologous KHP1 protein (Huang *et al.*, 1977; Kozminski *et al.*, 1995). Although *fla10-1* assembles normal-length flagella at the room temperature, it shortens and eventually loses flagella at 33°C. A culture of fully grown *fla10-1* cells was divided into two halves: one half was maintained at 25°C (room temperature) and the other half was treated at 33°C for 150 min. Their flagella were subsequently isolated and processed for Western blotting to determine the amounts of IFT139 (a component of IFT-A complex), IFT57 (a component IFT-B complex), and TTLL3 (Figure 6D). We found that along with IFT139 and IFT57, the signals of TTLL3 were clearly detected at room temperature but nearly undetectable at the sensitive temperature. This result indicates that the flagellar localization of TTLL3 is dependent on the IFT.

We surmised that majority of glycylation takes place during flagellar assembly. To investigate this, wild-type cells were exposed to pH-shock treatment and allowed a 30-min incubation to regenerate flagella (Figure 6E). We then isolated the regenerated flagella and the level of TTLL3 was compared with that in the steady-state flagella (Figure 5E). We discovered that, when normalized for the total protein content, the regenerated flagella contained approximately three times more TTLL3 than the steady-state flagella. However, given that the length of flagella that underwent 30-min regeneration is about half that of the steady-state flagella (Figure 1C), this finding implies that on a per flagellum basis, a regenerated flagellum has roughly 1.5 times more TTLL3 than a steady-state flagellum. Therefore, the frequency of TTLL3 transport increases during flagellar assembly.

### Tubulin glycylation plays a crucial role in the functioning of outer-arm dynein

**FIGURE 7:**
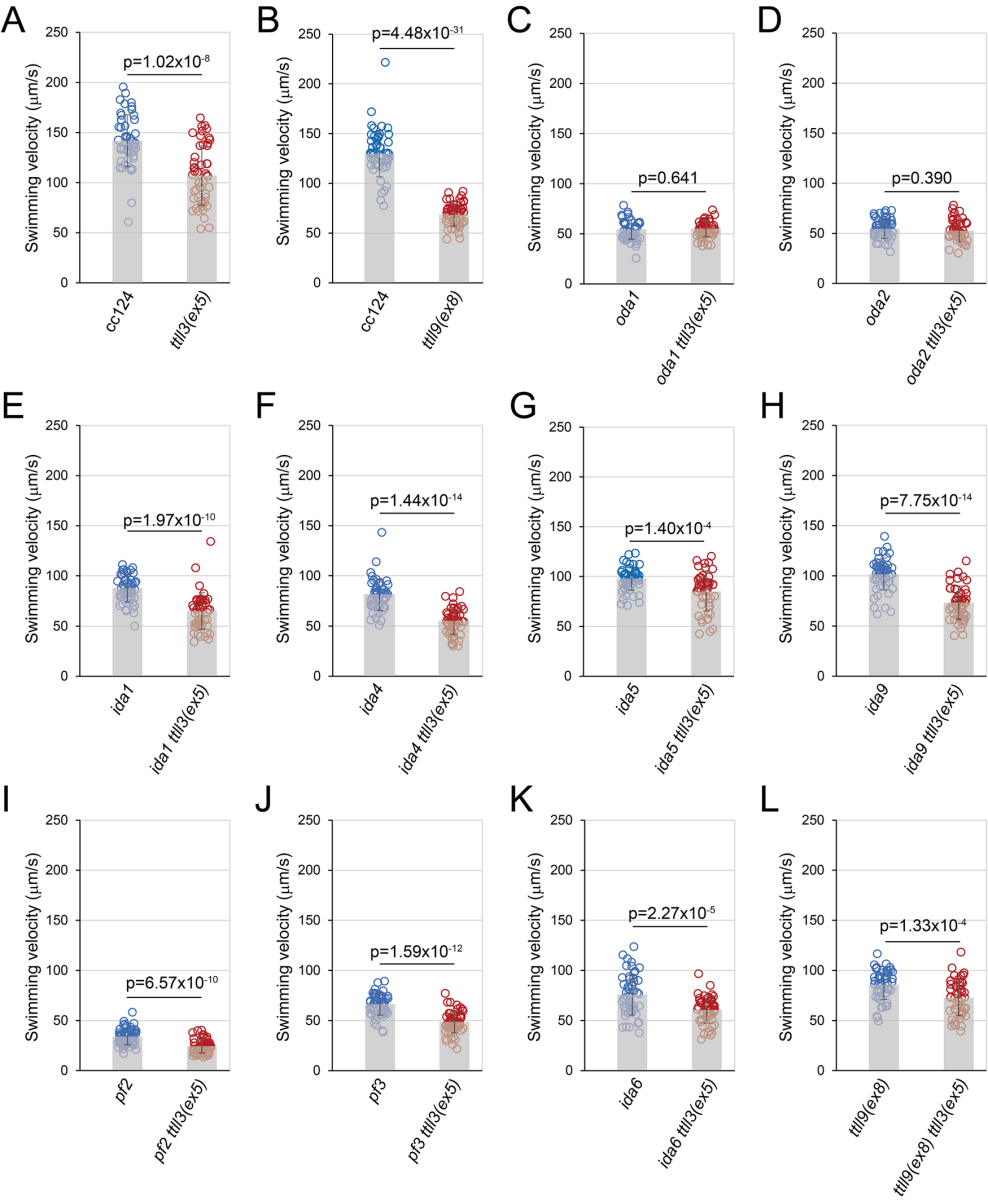
Swimming velocities of the *ttll3(ex5)* mutants Swimming velocities compared between (A) wild type (cc124) and *ttll3(ex5)*, (B) wild type (cc124) and *ttll9(ex8)*, (C) *oda1* and *oda1 ttll3(ex5)*, (D) *oda2* and *oda2 ttll3(ex5)*, (E) *ida1* and *ida1 ttll3(ex5)*, (F) *ida4* and *ida4 ttll3(ex5)*, (G) *ida5* and *ida5 ttll3(ex5)*, (H) *ida9* and *ida9 ttll3(ex5)*, (I) *pf2* and *pf2 ttll3(ex5)*, (J) *pf3* and *pf3 ttll3(ex5)*, (K) *ida6* and *ida6 ttll3(ex5)*, and (L) *ttll9(ex8)* and *ttll9(ex8) ttll3(ex5)*. Velocities were statistically evaluated by two-tailed unpaired Student’s *t* test.

Mouse sperm without tubulin glycylation show reduced swimming velocity (Gadadhar *et al.*, 2021), while the *Chlamydomonas* mutant *ttll3*, also generated through CRISPR/Cas9, displays increased swimming speed but decreased flagellar beat frequency (Viar *et al.*, 2023). The reason for the difference in the motility between these two organisms is currently unknown. To investigate the role of tubulin glycylation in ciliary motility, we conducted a comparative analysis of swimming motilities. First, we compared the swimming velocities of wild type (cc124) with those of *ttll3(ex5)*. We discovered that our *ttll3(ex5)* mutant tends to swim slower than the wild type (Figure 7A), although their swimming velocity was unstable varied depending on the cell concentration, temperature, and culture media. This finding seems to be inconsistent with the swimming speed of the *ttll3* mutant reported by Viar *et al.* (2023), although the wild-type strains used to generate the mutants were different. The reason for the observed differences in swimming behaviors of the *ttll3* strains across the two research groups are yet to be determined, highlighting the complexity of flagellar motility and the need for further investigation. Nevertheless, we decided to investigate the effect of glycylation on the motilities of various axonemal dynein lacking mutants.

To determine which axonemal dynein species is most significantly affected by glycylation, we assessed the effect of *ttll3(ex5)* in the background of a dynein-lacking mutation. If a particular dynein is affected by glycylation in the normal conditions, the effect of *ttll3(ex5)* mutation will not be observed when that dynein is absent. Axonemal dyneins are generally classified into two types; the outerarm dyneins, primarily involved in the force generation, and the innerarm dyneins, mainly involved in the waveform generation (Kamiya, 2002). In *Chlamydomonas*, the outer-dynein arms are composed of a single species comprising three heavy chains (α, β, and γ), whereas inner-dynein arms are composed of seven major species referred to as inner-arm dynein a to g. Interestingly, strains lacking the outer-arm dyneins, such as *oda1* and *oda2*, exhibited no discernible effects on motility from the *ttll3(ex5)* mutation (Figure 7, C and D). This suggests that tubulin glycylation mainly regulates the function of the outer-arm dyneins in the intact axoneme.

We then explored the effect of the *ttll3(ex5)* mutation in strains carrying a mutation that lacks the inner-arm dynein(s) (Figure 7, E, F, G, and H). *ida1* lacks inner-arm dynein f, *ida4* lacks inner-arm dynein a, c, and d, *ida5* lacks inner-arm dynein a, c, d, and e, and *ida9* lacks inner-arm dynein c. When combined with the *ttll3(ex5)* mutation, swimming velocities of all these mutants decreased, suggesting that tubulin glycylation does not affect the functions of inner-arm dynein a, c, d, e, and f. Contribution of glycylation to the other inner arm dyneins must be investigated in the future using mutants particularly lacking the inner arm dynein b and g.

We also found that the mutants lacking the N-DRC (*pf2*, *pf3*, and *ida6*) show reduced motility when combined with the *ttll3(ex5)* mutation, suggesting that glycylation does not affect the function of the N-DRC either (Figure 7, I, J, and K). Therefore, the swimming motilities of mutants lacking either the axonemal dyneins or N-DRC collectively show that tubulin glycylation plays an important role particularly in the functioning of the outer-arm dyneins.

We then explored the effect of glycylation in a mutant lacking polyglutamylation. We have previously demonstrated that tubulin polyglutamylation affects the function of the N-DRC using the mutant *tpg1*, which lacks TTLL9 responsible for elongating polyglutamate chains (Kubo *et al.*, 2010; 2012). As *tpg1*, the novel *ttll9(ex8)* mutant produced in the present study also showed reduced motility (Figure 7, B and L). We found that the double mutant *ttll9(ex8) ttll3(ex5)* swims more slowly than the mutant *ttll9(ex8)* alone (Figure 7L). This result suggests that the lack of glycylation additionally contributes to the decreased motility in the *ttll9(ex8)* flagella, suggesting a synergistic effect on flagella of lacking both modifications.

### Tubulin glycylation enhances sliding velocities of microtubule sliding in the disintegrated axonemes possessing the outer-arm dyneins

**FIGURE 8:**
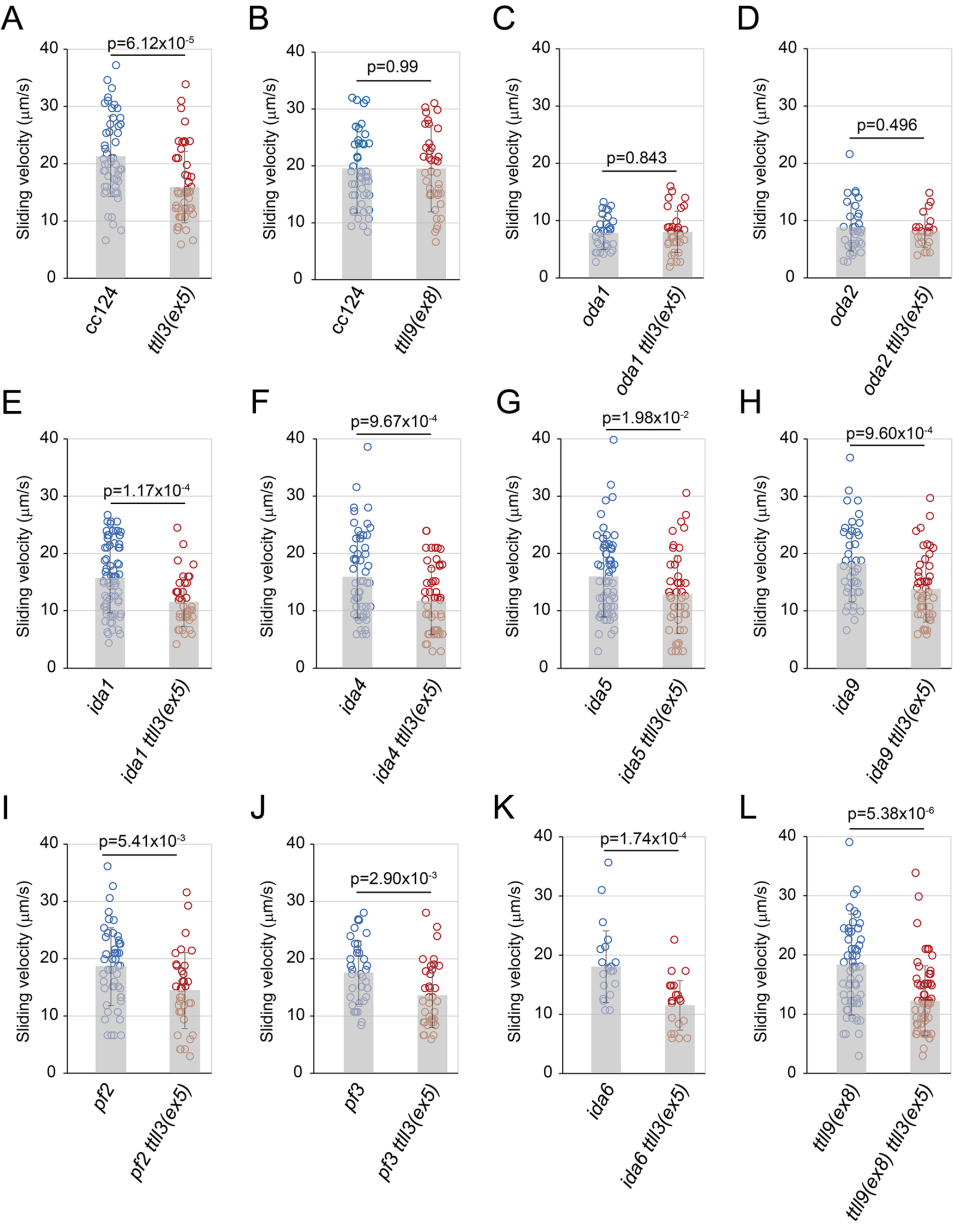
Sliding velocities of axonemal microtubules in the mutants combined with or without the *ttll3(ex5)* mutation Sliding velocities of axonemal microtubule compared between(A) wild type (cc124) and *ttll3(ex5)*, (B) wild type (cc124) and *ttll9(ex8)*, (C) *oda1* and *oda1 ttll3(ex5)*, (D) *oda2* and *oda2 ttll3(ex5)*, (E) *ida1* and *ida1 ttll3(ex5)*, (F) *ida4* and *ida4 ttll3(ex5)*, (G) *ida5* and *ida5 ttll3(ex5)*, (H) *ida9* and *ida9 ttll3(ex5)*, (I) *pf2* and *pf2 ttll3(ex5)*, (J) *pf3* and *pf3 ttll3(ex5)*, (K) *ida6* and *ida6 ttll3(ex5)*, and (L) *ttll9(ex8)* and *ttll9(ex8) ttll3(ex5)*. Velocities were statistically evaluated by two-tailed unpaired Student’s *t* test.

To further investigate the impact of the *ttll3(ex5)* mutation on ciliary motility, we assessed the sliding velocities of axonemal microtubules. We treated fragmented axonemes with ATP and protease to induce sliding disintegration. With 1 mM ATP treatment, the wild-type axonemes displayed microtubule sliding velocity of ∼20 μm/s. In contrast, the *ttll3(ex5)* axoneme showed a significantly reduced microtubule sliding velocity of ∼15 μm/s (Figure 8A). This finding suggests that glycylation enhances sliding of the axonemal microtubules. Importantly, in contrast to the axoneme possessing all set of the axonemal dyneins, the axonemes lacking the outer-arm dynein was not affected by the *ttll3(ex5)* mutation (Figure 8, C and D). These results confirm that glycylation specifically affects the function of the outer-arm dyneins.

We also compared axonemal microtubule sliding between wild type and *ttll9(ex8;* Figure 8B). Previous studies have demonstrated that the *tpg1* mutation lacking tubulin polyglutamylation significantly increases sliding velocity, particularly in axonemes lacking outer-arm dyneins, but to a lesser extent in intact axonemes possessing outer-arm dyneins (Kubo *et al.*, 2010; 2012). In the present study, the *ttll9(ex8)* axoneme showed almost the same sliding velocity compared with the wild-type axoneme (Figure 8B). This is consistent with the previous study using the same ATP concentration (Kubo *et al.*, 2010).

In contrast to the mutant axonemes lacking the outer-arm dyneins, however, mutant axonemes lacking the inner-arm dyneins (Figure 8, E, F, G, and H) and mutant axonemes lacking the N-DRCs (Figure 8, I, J, and K) all displayed significantly slower sliding velocities of axonemal microtubules when glycylation is absent. Furthermore, the *ttll9(ex8) ttll3(ex5)* axoneme displayed slower sliding velocity than *ttll9(ex8)* mutation alone, indicating that the lack of polyglutamylation does not recover the effect of *ttll3(ex8)* mutation (Figure 8L). Because glycylation predominantly occurs at the C-terminus region of β-tubulin in *Chlamydomonas* (Kubo *et al.*, 2023), these results indicate that axonemal tubulin glycylation enhances the sliding velocities of axonemal microtubule, presumably by neutralizing the minus charges arising from the glutamate residues in the β-tubulin C-terminus region.

## DISCUSSION

### Important roles of tubulin PTMs in cilia and flagella motility

In this study, we generated a novel *Chlamydomonas* mutant *ttll3(ex5)* to investigate the role of axonemal tubulin glycylation. The *ttll3(ex5)* strain exhibited slightly slower swimming velocity compared with the wild-type strain (Figure 7), indicating that tubulin glycylation influences flagellar motility, consistent with previous reports (Gadadhar *et al.*, 2021; Viar *et al.*, 2023). Our primary discovery is that tubulin glycylation affects the functions of the outer-arm dyneins likely by influencing the sliding velocities of the axonemal microtubules. This result is in sharp contrast to the ciliary role of tubulin polyglutamylation, another tubulin PTM, regulating the function of the N-DRC (Kubo *et al.*, 2012; Kubo and Oda, 2017).

### Specific pattern of tubulin glycylation in *Chlamydomonas*

Many organisms undergo polyglycylation both in α- and β-tubulin as determined by mass spectrometry. Specifically, *Paramecium* has a maximum of 19 and 18 glycine units on α- and β-tubulin (Redeker *et al.*, 1994; 2005); *Tetrahymena* has up to six and 12; sea urchin sperm have up to 11 and eight (Plessmann and Weber, 1997); and bull sperm have up to 23 and 15 (Multigner *et al.*, 1996). However, it is unlikely that “poly” glycylation occurs in *Chlamydomonas* (Kubo *et al.*, 2023) due to the absence of TTLL10, an enzyme essential for elongating glycine chains. The absence of polyglycylation is common to humans (Bré *et al.*, 1996) expressing inactivated TTLL10 (Rogowski *et al.*, 2009). Further, tubulin glycylation in *Chlamydomonas* is mostly confined to the glutamate residues in the C-terminus of β-tubulin, specifically at positions E435, E437, E439, E440, E441, and E442 (Kubo *et al.*, 2023). Considering that TTLL3 is a monoglycylase mainly for β-tubulin (Garnham *et al.*, 2017), the absence of α-tubulin glycylation in *Chlamydomonas* may be due to the lack of TTLL8, another monoglycylase. Elucidating the unique glycylation pattern of *Chlamydomonas*, such as determining the ratio of glycylated against unmodified β-tubulin, may present an intriguing challenge for future study. Also, because we have utilized only two types of antibodies, gly-pep1 and polyG (Kubo *et al.*, 2023), to investigate tubulin glycylation, employing additional antibodies such as AXO49 (recognizes monoglycylation) and TAP952 (recognizes polyglycylation; Bré *et al.*, 1996) may offer further insights.

Our finding suggests that glycylated tubulin is present at the outer-doublet microtubules and is absent from the central-pair microtubules (Figure 4). This observation contrasts with the findings of Orbach and Howard (2019), who employed a stepwise extraction method of axoneme to separate components of the central-pair from those of the outer-doublet microtubules. In the B-tubule outer doublets, glycylated tubulins are found in most of the protofilaments while polyglutamylated tubulins are present only at protofilament nine (Viar *et al.*, 2023). In the extraction procedure of the axoneme, components from the outer rim of the B-tubules sometimes are unintentionally contaminated to the central-pair microtubule fraction (Witman *et al.*, 1972). In Orbach and Howard (2019), this contamination may have led to the alternative interpretation that glycylated tubulin is localized to the central-pair microtubules.

It is noteworthy to mention that *Tetrahymena* has abundant glycylation in the outer doublet microtubules but also lacks glycylated tubulin in the central-pair microtubules (Wloga *et al.*, 2009). However, both the central-pair microtubules and the outer doublet microtubules are highly glycylated in *Drosophila* sperm. Therefore, there seems to be lineage-specific variations in the distribution of this specific tubulin modification. Additionally, in ciliates, glycylation is abundant on cortical microtubules (Xia *et al.*, 2000). These observations collectively suggest that tubulin glycylation serves additional functions beyond the regulation of ciliary motility.

### Dynamic transport of TTLL3 into cilia

We found that glycylation of axonemal tubulin occurs rapidly in the dikaryon produced between the wild-type and *ttll3(ex5)* gametes. We also found that recovery of glycylation in the dikaryon occurs evenly from the base to the tip of the cilia. These properties of modification share similarities to polyglutamylation, as we showed in our previous study (Kubo *et al.*, 2014), and was also confirmed in this study (Figure 5). Based on the cryoelectron microscopy presented by Viar *et al*. (2023), polyglutamylation is exclusive to protofilament nine of the B-tubule outer doublets, while glycylation occurs on the remaining protofilaments of the B-tubule. It is curious that TTLL3 and TTLL9, despite being needed to modify a different number of protofilaments, each recovered modification in the similar amount of time in the dikaryon. In vitro structural study showed that TTLL3 predominantly recognizes and glycylates four sites at the β-tubulin tail in a hierarchical manner (Garnham *et al.*, 2017). The mechanisms by which TTLL3 and TTLL9 identify their respective protofilaments in the axoneme remain unclear.

We observed a significant decrease in the amount of TTLL3 in the flagella of *fla10-1* when exposed to sensitive temperature. This indicates that flagellar recruitment of TTLL3 is dependent on the IFT (Figure 7D). Individual IFT proteins are often involved in the flagellar transport of particular cargoes. For example, IFT46, a core component of the IFT-B complex, plays a key role in the flagellar transport of outer-dynein arms (Hou *et al.*, 2007; Ahmed *et al.*, 2008). Another example is the N-termini of IFT74 and IFT81, which together transport tubulins into the flagella (Bhogaraju *et al.*, 2013; Brown *et al.*, 2015; Kubo *et al.*, 2016). Because a direct TTLL-IFT protein interaction remains unreported, investigating the interaction between TTLL3 and a specific IFT-particle protein remains a challenge for the future research.

### Tubulin glycylation impacts the function of outer-arm dyneins

By comparing motilities of various axonemal dynein mutants with or without the *ttll3(ex5)* mutation, we came up to the conclusion that tubulin glycylation affects the function of outer-arm dyneins. This is consistent with the previous study stating that tubulin polyglycylation affects the conformation change of axonemal dyneins including the outer arm dyneins (Gadadhar *et al.*, 2021). To further clarify whether glycylation specifically impacts certain dynein heavy chains or the entire outer-arm dynein complex, it would be interesting to examine double mutants combining the *ttll3* mutation with mutations in the alpha (*oda11*), beta (*oda4-s7*), and gamma (*oda2-t*) heavy chain motor units. Such experiments could provide precise insights into the specific dynein components most affected by tubulin glycylation. Additionally, to further explore this hypothesis, it may be necessary to perform in vitro motility assays using purified dyneins and polymerized microtubules that are reconstituted from the isolated axonemes. The *ttll3(ex5)* mutants generated in this study will value such experiments in the future.

In *Chlamydomonas*, glycylation primarily occurs at the six glutamate residues in the C-terminal region of β-tubulin (Kubo *et al.*, 2023) specifically through the attachment of glycine residues to the γ-carboxyl groups of these glutamates (Redeker *et al.*, 1994). This occurrence of glycylation may reduce the local negative charges originating from the γ-carboxyl groups of the glutamate residues. Our sliding disintegration assay of the isolated axonemes supports this concept. We found that in glycylation-deficient axonemes, microtubule sliding is significantly slower, especially in the presence of outer-arm dyneins (Figure 8). Given that the negative charges in the tubulin C-terminal regions are thought to enhance the interactions between microtubules and dyneins in the axoneme (Kubo *et al.*, 2010), the reduced sliding speed is likely due to stronger electrostatic interactions between the microtubules.

We were unable to reason the discrepancy of the swimming velocities of the *ttll3* mutants between ours and those of Viar *et al.* (2023). However, we noticed that the motilities of *ttll3(ex5)* is relatively sensitive, being affected by varying culture conditions such as media, cell concentration, temperature, pH, and light. This suggests that the absence of glycylation largely affect the electrostatic interactions between outer-doublet microtubules and outer-arm dyneins under certain conditions, leading to variations in motility. The precise impact of glycylation on these interactions remains a compelling direction for future research, promising to deepen our understanding of the molecular mechanisms governing cellular movement.

## MATERIALS AND METHODS

Request a protocol through *Bio-protocol*.

### Strains and Cultures

The strains (Supplemental Table 1) were maintained on Tris-acetate-phosphate (TAP; Gorman and Levine, 1965) agar plates for long-term storage. For the experiments, the cells were cultured in liquid TAP medium at 25°C with a light/dark cycle of 12:12 h and aeration.

### Generation of CRISPR/Cas9 mutants

The *ttll3(ex5)* and *ttll9(ex8)* mutant strains were generated following the method outlined in Kubo *et al.* (2023). The target sequences were identified using CRISPRdirect (http://crispr.dbcls.jp/). The crRNA (Supplemental Table 2) and tracrRNAs (fasmac) were dissolved in Nuclease-Free Duplex Buffer (Integrated DNA technologies (IDT) buffer).

To generate the guide RNA, 1 μl of crRNA (40 μM) was annealed with 1 μl of tracrRNA (40 μM) by heating the mixture to 95°C for 2 min and then gradually cooling it down to 25°C over 90 min. Subsequently, 2 μl of annealed guide RNA was combined with 0.5 μl of Cas9 protein (10 μg/ml; fasmac) and 7.5 μl IDT buffer, followed by incubation at 37°C for 15 min to produce Cas9/gRNA RNP.

The cells were treated with autolysin for 60 min while being gently rocked, followed by a gentle agitation at 40°C for 30 min. After centrifugation (2000 rpm, 3 min, at room temperature), the cells were collected and subjected to electroporation using the Cas9/gRNA RNP (10 μl) and the donor DNA (10 μl; up to 2 μg). The transformed cells were suspended in 10 ml of TAP sucrose medium and gently agitated for 24 h under low light conditions. Cells were then collected by centrifugation (2000 rpm, 3 min, at room temperature), plated on TAP-agar with 10 μg/ml hygromycin or 10 μg/ml paromomycin, and cultured for 5–7 d under continuous light. Colonies were isolated into a 96-well plate and cultured for several days. The TTLL3 mutation was confirmed using the primer set TTLL3-15-F (GCGGAATGAGCGCGGAAACAGTCAC) and TTLL3-15-R (CGTCCACCGCACGTCCTGGAAGAAC); and the TTLL9 mutation was confirmed using the primer set TTLL9-2-F (GACATGTATTCCTCTCTCTCGTGAC) and TTLL9-2-R (TCCTTCAACTAAAAATGGTGAAGAC) to check for insertion mutations.

### Measurements of flagellar length

To measure flagellar regeneration kinetics, cells were first deflagellated by adjusting the pH of the liquid culture to 4.5 using 0.5 M CH_3_COOH. This was followed by a brief exposure period of 20 s. Subsequently, the pH was neutralized to 7.4 with 0.5 M KOH to facilitate a 180-min incubation period. At 15-min intervals, aliquots of the cells were collected and fixed by adding glutaraldehyde to achieve a final concentration of 0.3%.

To measure LiCl-induced flagellar elongation, 1 M Lithium Chloride (Nakalai Tesque) was added to the cell culture to obtain a final concentration of 25 mM. Aliquots of cells were collected every 30 min and fixed by 0.3% glutaraldehyde.

To measure NaPPi-induced flagellar shortening, 0.2 M Sodium Pyrophosphate (Sigma) was added to the cell culture to obtain a final concentration of 25 mM. Aliquots of cells were collected every 30 min and fixed by 0.3% glutaraldehyde.

For each flagellar length measurements, the fixed cells were concentrated through centrifugation at 2500 rpm for 1 min. Subsequently, ∼ 5 µl of the cells were embedded on a slide and observed under a microscope (BX53, Olympus) equipped with a 20 × UPlan FL N objective lens (Olympus). Images were captured using a CCD camera (ORCA-Flash4.0 V3 sCMOS, Hamamatsu). The images were analyzed by ImageJ, and the lengths of at least 50 flagella from individual cells were measured to obtain average length and SD.

### Indirect immunofluorescence microscopy

Immunostaining of NFAps was carried out following Sanders and Salisbury (1995). Fully grown cells were suspended into 6 ml autolysin and gently agitated for 60 min to remove cell walls. The cells were washed with NB buffer (6.7 mM Tris-HCl [pH 7.2], 3.7 mM EGTA, 10 mM MgCl_2_, and 0.25 mM KCl) and placed on an eight well-slide glass (8-mm well; Matsunami) treated with polyethylenimine. The cells were demembranated by 1% Igepal CA-630 (Sigma) and subsequently fixed with 2% paraformaldehyde in NB buffer for 10 min. The cells were then treated with acetone at -20°C and methanol at -20°C for 5 min each. The cells were first treated with blocking buffer (1% bovine serum albumin and 3% Fish Skin gelatin in phosphate-buffered saline [PBS]) and incubated with primary antibodies (Supplemental Figure 3) followed by secondary antibodies (goat antirabbit IgG Alexa 488, 1:200, Invitrogen; goat antimouse IgG Alexa Fluor 594, 1:200, Invitrogen). The cells were treated with antifade mountant (SlowFade Diamond, Thermo Fisher Scientific) and encapsulated with a glass coverslip. The sample was examined with a microscope (BX53, Olympus) equipped with an objective lens (100 × UPlan FL N Oil objective, Olympus). Images were acquired with a CCD camera (ORCA-Flash4.0 sCMOS, Hamamatsu).

### Isolation of axoneme

Cilia were isolated according to Witman *et al.* (1972) with some modifications. Briefly, fully grown cells were collected with centrifugation (3000 rpm, 5 min) and treated with 1 mM dibucaine-HCl (Wako) to amputate their cilia. Cilia were washed and collected by centrifugation (15,000 rpm, 20 min, 4°C). Isolated cilia were suspended into HMDEK buffer (30 mM HEPES, 5 mM MgSO4. 1 mM DTT, 0.1 mM EGTA, and 25 mM CH3COOK) and demembranated by 0.1% Igepal CA-630 (Sigma) to prepare axonemal samples. For the immunofluorescence microscopy of the axonemal microtubules, isolated axonemes were placed on eight-well slide glass and treated with 0.1 mM ATP (Sigma) and 0.5 μg/ml Type VII bacterial protease (Sigma) to induce splaying. Immunostaining of the splayed axonemes were processed as described in the “Indirect immunofluorescence microscopy” section.

### Western blotting

Western blot analysis was conducted as described by Towbin *et al.* (1979) with minor adjustments. SDS-protein samples were electrophoretically separated using handmade SDS-polyacrylamide gels (7.5 or 9% polyacrylamide) and then transferred onto a PVDF membrane (Immobilon-P, pore size 0.45 μm; Merck-Millipore). The membrane was probed with primary antibodies (Supplemental Figure 3), followed by secondary antibodies: goat antimouse IgG (H+L; Invitrogen, Catalogue#31430) or goat antirabbit IgG (H+L; Invitrogen, Catalogue#31460). After Chemi-Lumi One Super treatment (Nakalai Tesque, #02230), the membrane was visualized using the Fusion Solo S system (Vilver Bio Imaging).

### Generation of the anti-TTLL3 antibodies

Two types of peptide antibodies were custom-synthesized by Bio-Synthesis (www.biosyn.com/). Peptides of N-terminus region (DSTKVNDSGAKEKSWADRNK-Cys) and C-terminus region (Cys-GLRAKTSSPIRMRTPRLSS) of TTLL3 were synthesized. The peptides were conjugated to keyhole limpet hemocyanin (KLH) to enhance immunogenicity before being injected into rabbits. The immunization protocol followed a standard procedure provided by Bio-synthesis, consisting of an initial immunization followed by three to six booster injections. Serum was collected after the final booster, and antibody titers were assessed by enzyme-linked immunosorbent assay (ELISA) using the synthesized peptides as antigens. Antibodies were purified using affinity chromatography with the corresponding peptide sequences immobilized on the column. The antibodies were eluted from the column using 0.1 M glycine-HCl (pH 2.8) and then neutralized with Tris-HCl (pH 8.0) buffer. The buffer was subsequently replaced with PBS at pH 7.4 via dialysis, and NaN_3_ was added to a final concentration of 0.02%. The purified antibodies were then tested for specificity and sensitivity by Western blotting using cell lysates and flagellar proteins.

### Assessment of motility

Swimming velocity was acquired by tracking images of the moving cells. Briefly, the cells under the dark-field microscope equipped with 40x objective were recorded using a digital camera with a frame rate of 30 fps, and the obtained movies were processed with ImageJ.

Microtubule sliding velocity during axonemal disintegration was measured as described previously (Kurimoto and Kamiya, 1991). Briefly, fragmented axonemes (∼5 μm in length) were placed in a perfusion chamber under a dark-field microscope. Microtubule sliding was induced by a solution containing 0.5 μg/ml Type VII bacterial protease (Sigma) and ATP (Sigma). This process was recorded using a × 100 objective, an oil-immersion dark-field condenser, a light source of a mercury lamp (Olympus, U-RLF-T), and a CCD camera with a frame rate of 30 fps (Olympus, M-3204C).

## Supplementary Material



## Abbreviations used:

ATP: adenosine triphosphate
CBB: coomassie brilliant blue
CCP: cytosolic carboxypeptidase
CRISPR: clustered regularly interspaced short palindromic repeats
IFT: intraflagellar transport
LiCl: lithium chloride
*N-DRC*: nexin-dynein regulatory complex
NaPPi: sodium pyrophosphate
NFAp: nuclear-flagellar apparatus
PTM: posttranslational modification
TMCP: tubulin metallocarboxypeptidase
TTLL: tubulin tyrosine ligase-like
