# Supplementary material for: Tubulin glycylation controls ciliary motility through modulation of outer-arm dyneins
Source: Mol Biol Cell. 2024 Jul 1;35(7):ar90. doi: 10.1091/mbc.E24-04-0154 (PMC11244163; doi:10.1091/mbc.E24-04-0154)
Supplement: Supplementary file 1 [file mbc-35-ar90-s001.pdf]

Supplemental Materials

Molecular Biology of the Cell

Kubo *et al*.

Supplemental Data:

Tubulin glycylation controls ciliary motility through modulation of outer-arm dyneins

Tomohiro Kubo[1]*, Rinka Sasaki[1], and Toshiyuki Oda[1*]
[1]Department of Anatomy and Structural Biology, Graduate School of Medicine, University of Yamanashi, 1110 Shimokato, Chuo, Yamanashi, 409-3898, Japan
*Corresponding authors
Email addresses: tkubo@yamanashi.ac.jp; toda@yamanashi.ac.jp

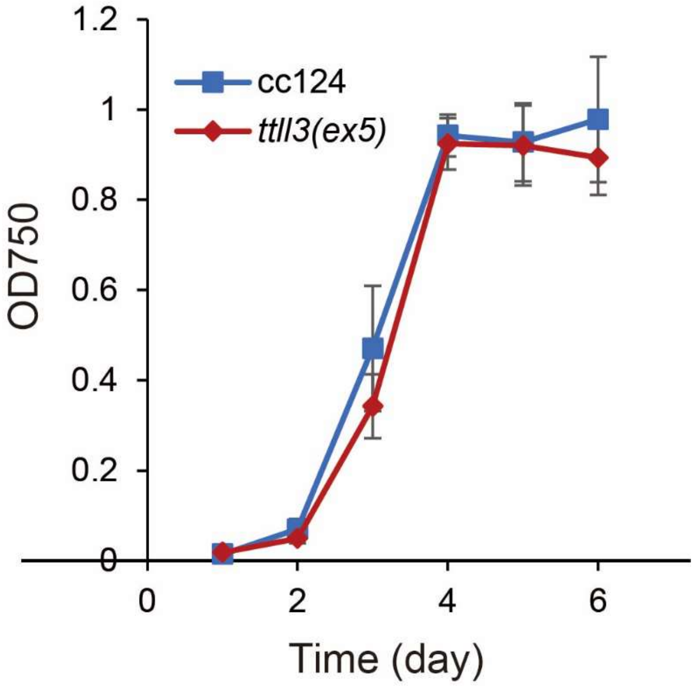

Supplemental Figure 1. The *ttll3(ex5)* mutant proliferates normally
The proliferation rates of wild type (cc124) and *ttll3(ex5)*. Cell densities were assessed by measuring the daily OD750. The average values, along with standard deviations, were presented for each measurement based on three independent samples.

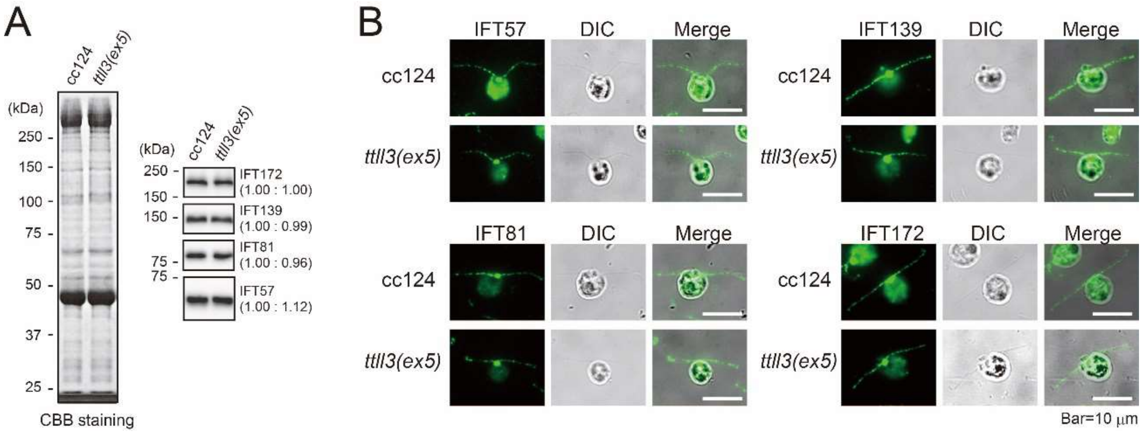

Supplemental Figure 2. IFT amounts and localizations are normal in the *ttll3(ex5)* flagella

(A) CBB-stained gel and Western blotting of wild-type (cc124) and *ttll3(ex5)* flagella. Antibodies against IFT172, IFT139, IFT81 and IFT57 were used. The quantification of the band intensities, normalized to those of the tubulin bands in the CBB stained gel, is shown in brackets, and are based on three experiments. (B) Immunostaining of cell bodies of wild type (cc124) and *ttll3(ex5)* stained with IFT172, IFT139, IFT81 and IFT57 antibodies.

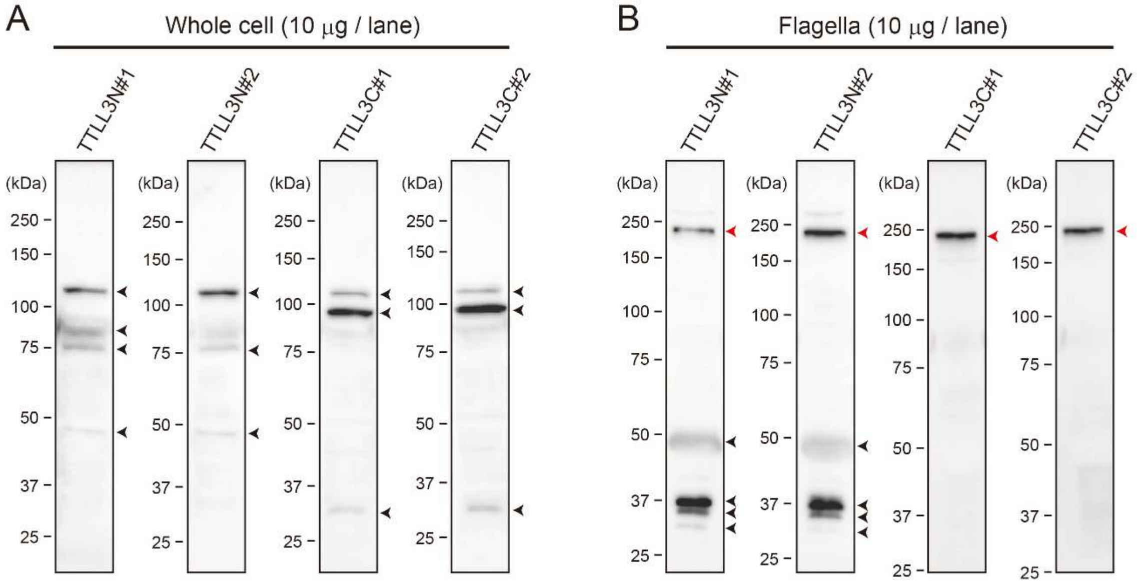

Supplemental Figure 3. Characterization of novel anti-TTLL3N and TTLL3C antibodies

Western blotting of (A) whole-cell and (B) flagellar samples was performed using anti-TTLL3N#1, TTLL3N#2, TTLL3C#1, and TTLL3C#2 antibodies. Non-specific bands are indicated by black arrowheads, while TTLL3 bands are indicated by red arrowheads. The data in (B) are partially presented in the main text (see Figure 6A).

Supplemental Table 1. List of strains used in this study

**Name**	**Origin**	**Mating type**	**Antibiotic resistance**
cc124		Minus	
cc125		Plus	
ttll3(ex5)	cc124	Minus	Hygromycin
ttll9(ex8)	cc124	Minus	Paromomycin
ttll9(ex8) ttll3(ex5)	*ttll3(ex5)-*	Minus	Hygromycin, paromomycin
oda1	cc2229	Minus	
oda1 ttll3(ex5)	cc2229	Minus	Hygromycin
oda2	cc2231	Minus	
oda2 ttll3(ex5)	cc2231	Minus	Hygromycin
ida1	cc2665	Minus	
ida1 ttll3(ex5)	cc2665	Minus	Hygromycin
ida4	cc2670	Plus	
ida4 ttll3(ex5)	cc2670	Plus	Hygromycin
ida9	cc4075	Minus	
ida9 ttll3(ex5)	cc4075	Minus	Hygromycin
pf2	cc513	Minus	
pf2 ttll3(ex5)	cc513	Minus	Hygromycin
pf3	cc604	Minus	
pf3 ttll3(ex5)	cc604	Minus	Hygromycin
ida6	cc3091	Minus	
ida6 ttll3(ex5)	cc3091	Minus	Hygromycin

*Generated mutants will be deposited to the *Chlamydomonas* Resource Center (https://www.chlamycollection.org/) after publication.

Supplemental Table 2. List of crRNA sequences

crRNAs	Sequence 5′-3′	Description
TTLL3 (ex5)	CCGCAGGGCGUAUGACAUCUguuuuagagcuaugcuguuuug	For TTLL3 knockout
TTLL9 (ex8)	GCGCGCGGAUGAUGAUGUUCguuuuagagcuaugcuguuuug	For TTLL9 knockout

Supplemental Table 3. Antibody used in this study

**Antibody (clone #)**	**Host**	**Dilution**	**Reference / Origin**
Anti-glycylated tubulin (Gly-pep1)	Rabbit	1:2000 (WB), 1:200 (IFM)	Funakoshi
Anti-polyglutamylated tubulin (polyE#2)	Rabbit	1:2000 (WB), 1:200 (IFM)	Kubo et al. (2017)
Anti-glutamylated tubulin (GT335)	Mouse	1:2000 (WB), 1:200 (IFM)	Adipogen Life Sciences
Anti-detyrosinated tubulin (AB3201)	Rabbit	1:2000 (WB)	Merck
Anti-tyrosinated tubulin (TUB-1A2)	Mouse	1:3000 (WB)	Sigma-Aldrich
Anti-acetylated tubulin (6-11B-1)	Mouse	1:5000 (WB)	Sigma-Aldrich
Anti-α-tubulin (B512)	Mouse	1:5000 (WB)	Sigma-Aldrich
Anti-IFT172	Mouse	1:20 (WB)<br>1:2 (IFM)	DSHB
Anti-IFT139	Mouse	1:100 (WB)<br>1:10 (IFM)	DSHB
Anti-IFT81	Mouse	1:100 (WB)<br>1:10 (IFM)	DSHB
Anti-IFT57	Mouse	1:20 (WB)<br>1:2 (IFM)	DSHB
Anti-TTLL3N#1	Rabbit	1:2000 (WB)	This study
Anti-TTLL3N2#2	Rabbit	1:2000 (WB)	This study
Anti-TTLL3C#1	Rabbit	1:2000 (WB)	This study
Anti-TTLL3C2#2	Rabbit	1:2000 (WB)	This study
